# Supplementary material for: Taxonomic and Functional Characteristics of the Gill and Gastrointestinal Microbiota and Its Correlation with Intestinal Metabolites in NEW GIFT Strain of Farmed Adult Nile Tilapia (Oreochromis niloticus)
Source: Microorganisms. 2021 Mar 17;9(3):617. doi: 10.3390/microorganisms9030617 (PMC8002438; doi:10.3390/microorganisms9030617)
Supplement: Supplementary file 1 [file microorganisms-09-00617-s001.pdf]

Supplementary information to the article:

Taxonomic and functional characteristics of the gill and gastrointestinal microbiota and its correlation with intestinal metabolites in NEW GIFT strain of farmed adult Nile tilapia (*Oreochromis niloticus*)

Zhenbing Wu[1,2], Qianqian Zhang[1,3], Yaoyao Lin[1,2], Jingwen Hao[1,2], Shuyi Wang[1,2], Jingyong Zhang[1,3], Aihua Li[1,3]*

[1] State Key Laboratory of Freshwater Ecology and Biotechnology, Institute of Hydrobiology, Chinese Academy of Sciences, Wuhan 430072, China; wuzhenbing@ihb.ac.cn (Z.W.); zqq@ihb.ac.cn (Q.Z.); linyaoyao@ihb.ac.cn (Y.L.); haojingwen@ihb.ac.cn (J.H.); wangsy@ihb.ac.cn (S.W.); zhangjy@ihb.ac.cn (J.Z.)

[2] University of Chinese Academy of Sciences, Beijing 100049, China

[3] Key Laboratory of Aquaculture Disease Control, Ministry of Agriculture, P. R. China

* Correspondence: liaihua@ihb.ac.cn; Tel.: 86-27-68780053

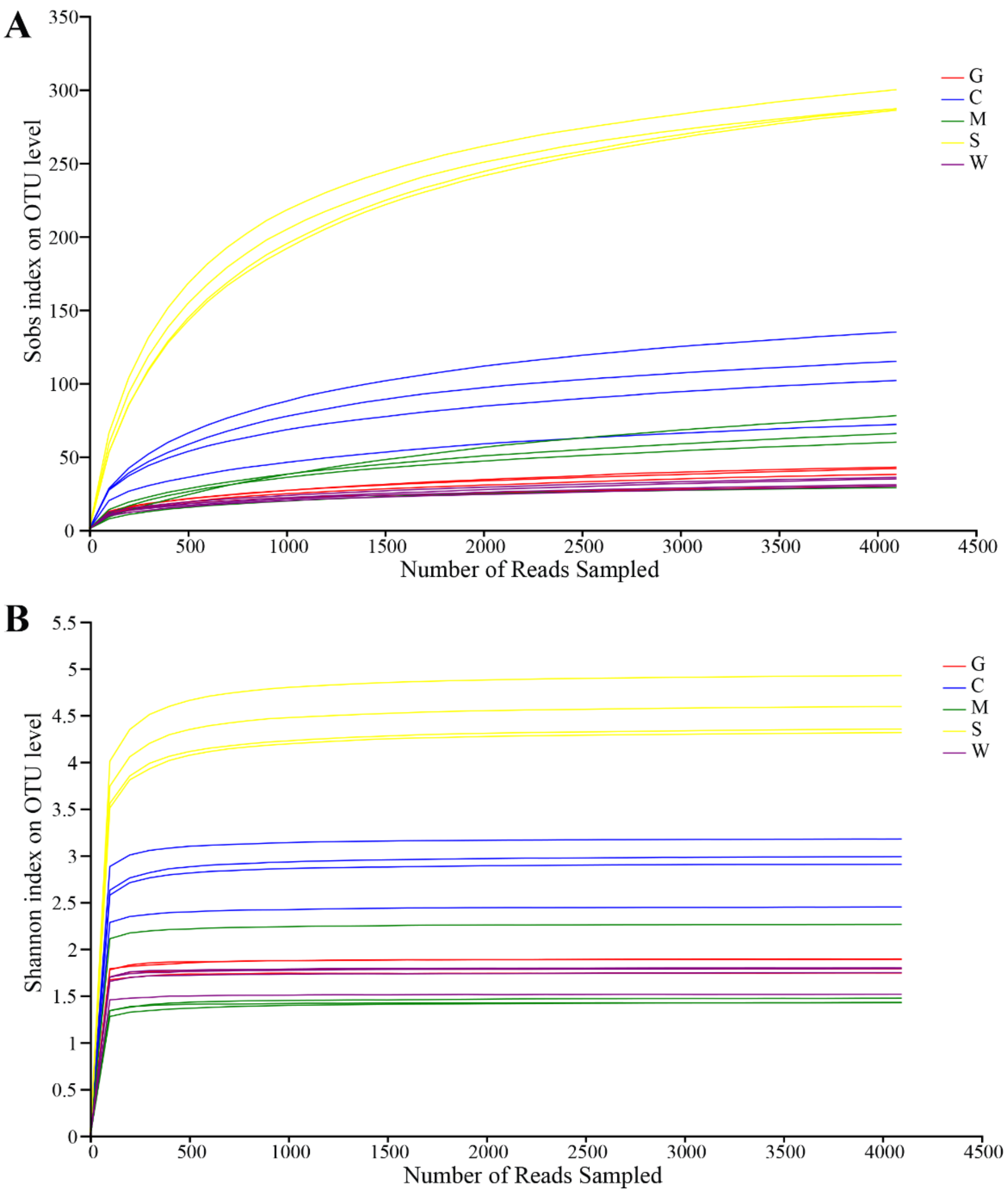

Figure S1. Rarefaction analysis of OTUs clustered at 97% sequence identity of all twenty samples. (A) Rarefaction curves on OTU level of all samples; (B) Shannon curves on OTU level of all samples. G: gill mucosae (G1-G4), C: intestinal contents (C1-C4), M: intestinal mucosae (M1-M4), S: stomach contents (S1-S4), W: stomach mucosae (W1-W4).

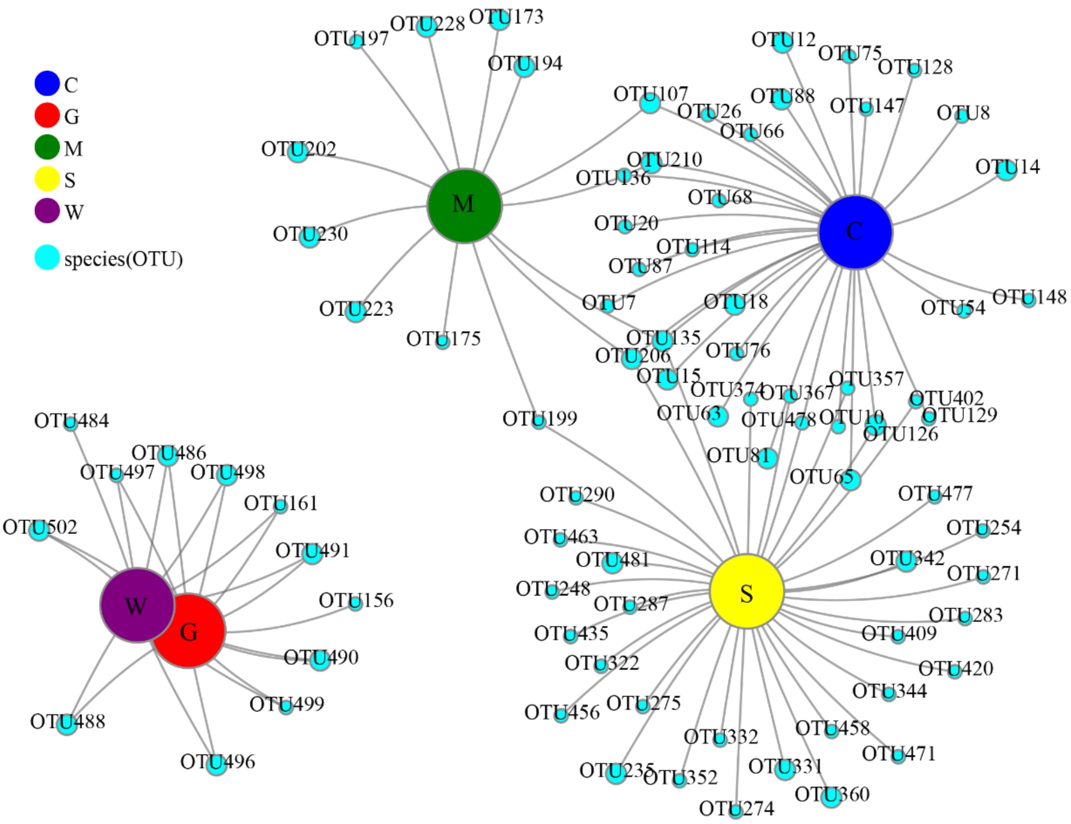

Figure S2. The co-occurrence network at the OTU level (average relative abundance > 0.5%) among five different sites. In the network, sites were colored by large nodes (Gill mucosae = red; intestinal contents = blue; intestinal mucosae = green; stomach contents = yellow; stomach mucosae = purple). Small purple nodes represent the OTUs. Lines connecting a site to an OTU indicate that the OTU is observed in that site.

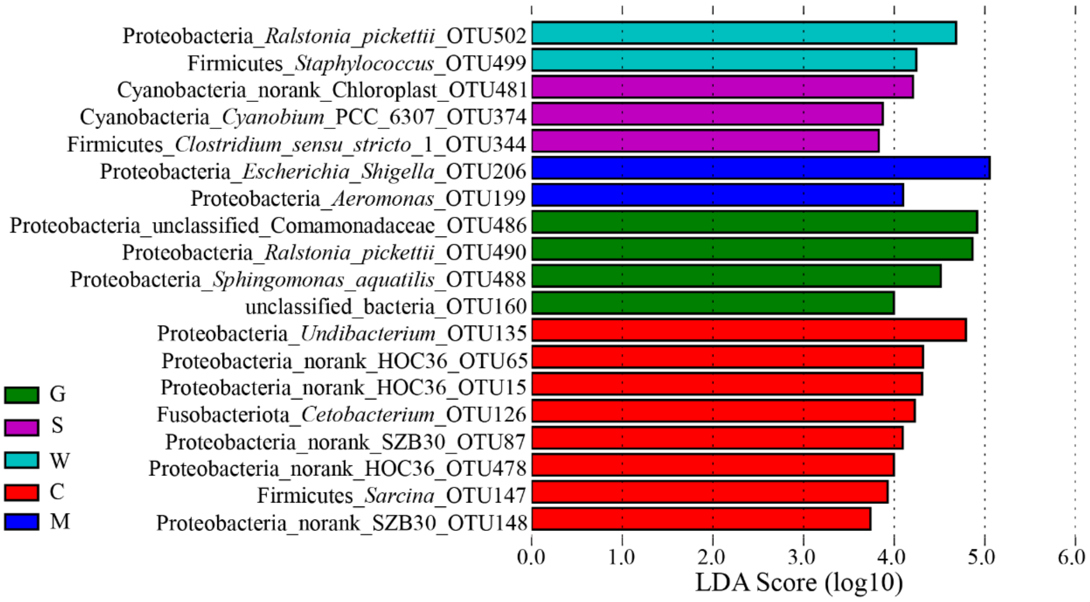

Figure S3. LEfSe showing differences in the bacterial communities at the OTU level among five different sites. The highlighted taxa are enriched in the group that corresponds to each color. LDA scores can be interpreted as the degree of difference in the relative abundance of OTUs. G: gill mucosae (G1-G4), C: intestinal contents (C1-C4), M: intestinal mucosae (M1-M4), S: stomach contents (S1-S4), W: stomach mucosae (W1-W4).

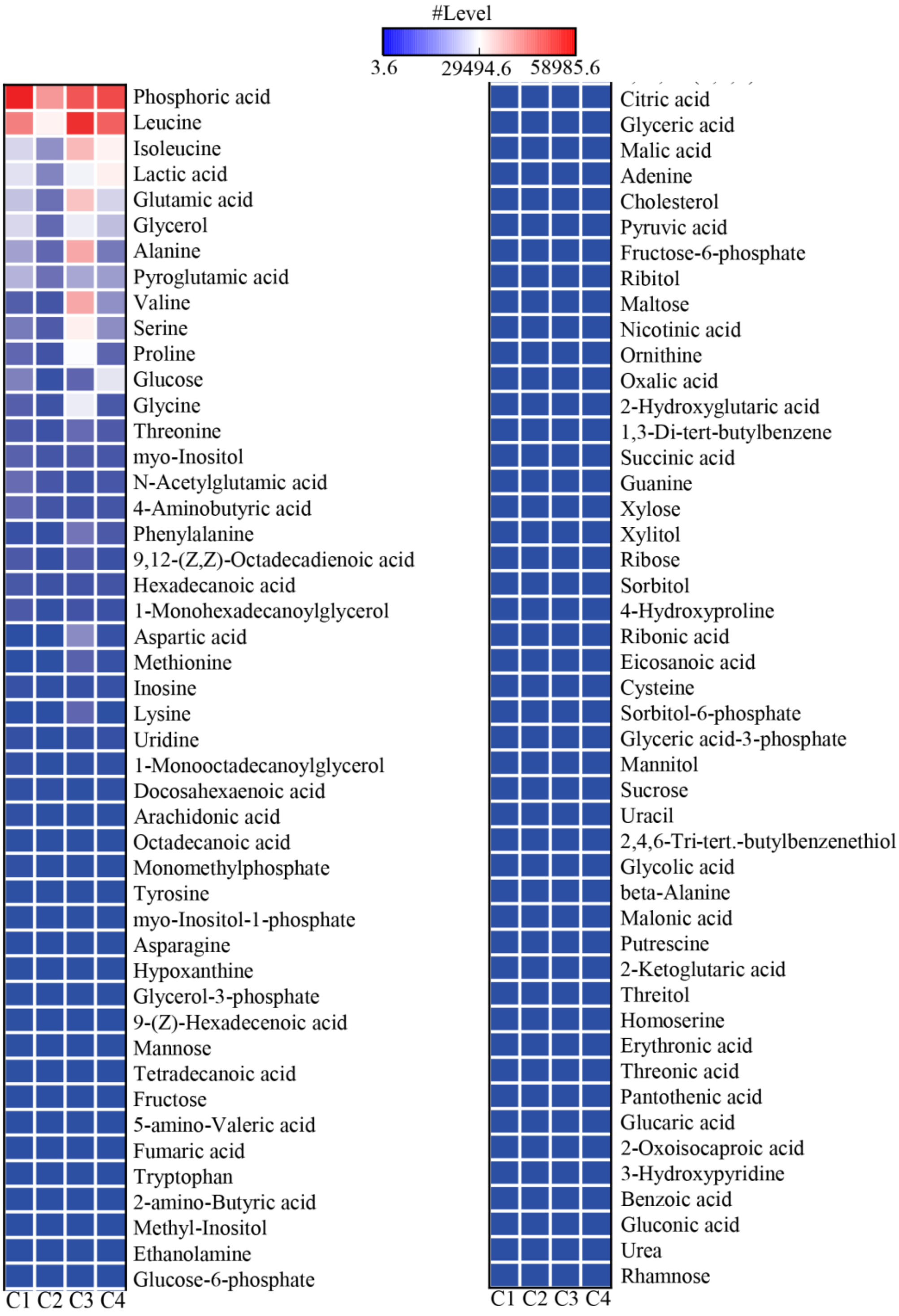

Figure S4. Heatmap profiles showing the 95 different intestinal metabolites detected in the intestinal contents using GC/MS analysis. Rows represent the intestinal metabolites, columns represent the intestinal content samples, and the color intensity in the heatmap represents the abundance of intestinal metabolites. C: intestinal contents (C1-C4).

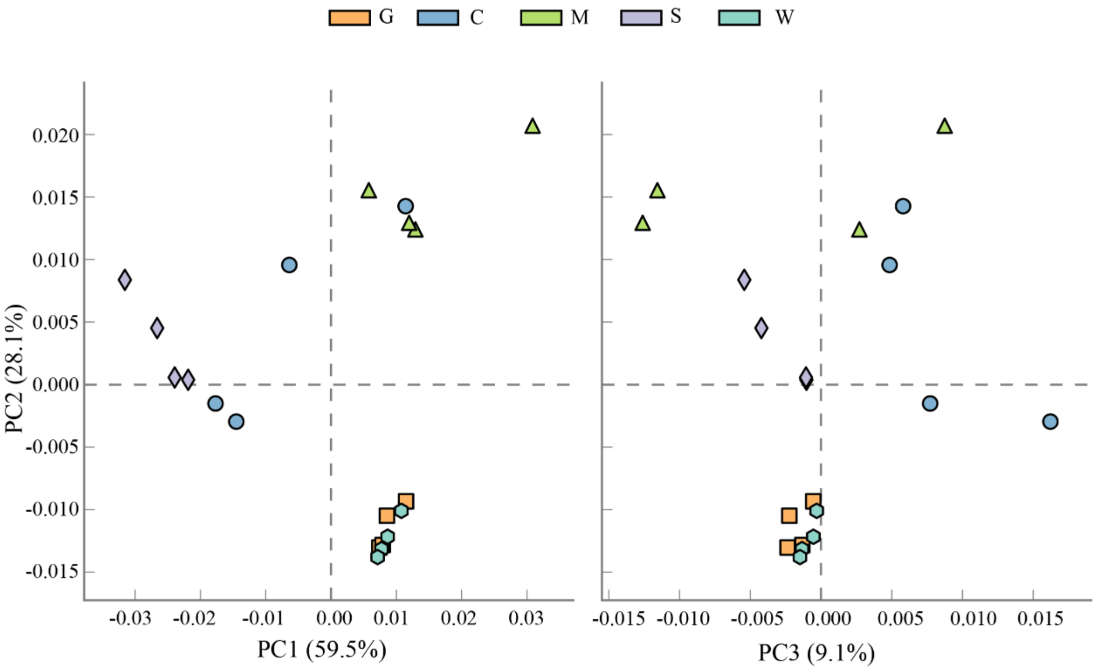

Figure S5. Principal Component Analysis (PCA) visualizing the integral structure dissimilarities of microbial function among five different sites. G: gill mucosae (G1-G4), C: intestinal contents (C1-C4), M: intestinal mucosae (M1-M4), S: stomach contents (S1-S4), W: stomach mucosae (W1-W4).

Table S1. Summary of alpha diversity indices and the number of taxa calculated based on a cutoff of 97% similarity of 16S rRNA sequences in different sites.

Indices	Group				
	G	C	M	S	W
OTUs	38.25 ± 5.91	106.00 ± 26.42	58.25 ± 20.89	290.00 ± 6.68	33.00 ± 2.94
Chao 1	49.09 ± 11.61	124.13 ± 29.21	76.91 ± 33.08	332.48 ± 14.20	50.08 ± 10.95
Shannon	1.83 ± 0.07	2.88 ± 0.31	1.65 ± 0.41	4.55 ± 0.28	1.71 ± 0.13
Simpson	0.25 ± 0.02	0.12 ± 0.04	0.31 ± 0.11	0.03 ± 0.01	0.28 ± 0.04
Number of phyla	10	15	13	24	9
Number of classes	14	33	23	55	13
Number of orders	29	76	53	103	29
Number of families	40	90	67	146	38
Number of genera	48	111	88	201	46
Number of OTUs	67	187	132	373	61
Coverage	99.74 ± 0.07%	99.41 ± 0.11%	99.52 ± 0.27%	98.74 ± 0.13%	99.75 ± 0.05%

Table S2. Analysis of similarity (ANOSIM) of the structure and function of the bacterial communities at different sites based on the Bray-Curtis metric. Permutation N = 999; R is assessed by permuting the grouping vector to obtain the empirical distribution of R under the null model; a *p*-value less than 0.05 means significant.

Group	Community structure		Community function	
	R	*p*	R	*p*
Whole comparison	0.8375	0.001	0.7267	0.001
G vs. C	1	0.034	0.8125	0.031
G vs. M	1	0.034	0.7708	0.029
G vs. S	1	0.034	1	0.033
G vs. W	-0.0417	0.497	-0.0938	0.614
C vs. M	0.8333	0.034	0.5729	0.016
C vs. S	0.9531	0.034	0.6667	0.026
C vs. W	1	0.034	0.8125	0.037
M vs. S	1	0.034	1	0.039
M vs. W	1	0.034	0.7917	0.029
S vs. W	1	0.034	1	0.026

Table S3. Comparison of dominant phyla (> 1%, average relative abundance in all samples) in the bacterial communities of different sites. The same letter indicates no significant differences ($p > 0.05$) between the two groups, while the different letter indicates significant differences ($p < 0.05$ or $p < 0.01$) between the two groups.

Phyla	Group				
	G	C	M	S	W
Proteobacteria	95.25 ± 1.08% a	59.69 ± 13.36% b	80.16 ± 11.98% ab	33.00 ± 3.42% c	95.35 ± 0.56% a
Actinobacteriota	2.81 ± 0.40% a	24.21 ± 12.67% b	0.56 ± 0.44% c	0.40 ± 0.16% c	2.70 ± 0.40% a
Bacteroidota	0.02 ± 0.04% a	0.09 ± 0.09% a	0.05 ± 0.10% a	27.88 ± 3.29% b	0.05 ± 0.04% a
Firmicutes	0.91 ± 0.59% a	7.63 ± 7.00% a	11.80 ± 7.67% a	1.51 ± 0.41% a	1.35 ± 0.40% a
Cyanobacteria	0.04 ± 0.05% ab	0.93 ± 0.63% c	0.21 ± 0.14% ac	12.36 ± 7.37% d	0.01 ± 0.02% b
Fusobacteriota	0.02 ± 0.02% a	3.50 ± 4.95% b	3.52 ± 6.38% b	2.72 ± 1.04% b	0.01 ± 0.01% a
Verrucomicrobiota	0.06 ± 0.07% a	0.18 ± 0.17% a	0.02 ± 0.02% a	7.67 ± 6.10% b	0.01 ± 0.01% a
Chloroflexi	0 a	1.13 ± 0.66% b	0.07 ± 0.12% a	4.98 ± 2.16% c	0 a

Table S4. The ten highest average abundant OTUs in the bacterial community of five different sites

G	C	M	S	W
Sphingomonas aquatilis OTU498 (42.95 ± 6.35%)	*Undibacterium* OTU135 (11.95 ± 16.02%)	*Escherichia-Shigella* OTU206 (21.12 ± 3.59%)	Chitinophagaceae sp. OTU342 (9.84 ± 2.41%)	*Sphingomonas aquatilis* OTU498 (44.19 ± 8.87%)
Comamonadaceae sp. OTU486 (16.29 ± 5.02%)	Propionibacteriaceae sp. OTU18 (11.85 ± 15.23%)	*Undibacterium* OTU210 (19.25 ± 24.65%)	Verrucomicrobiae sp. OTU360 (5.95 ± 5.97%)	*Ralstonia pickettii* OTU490 (12.08 ± 12.16%)
Ralstonia pickettii OTU490 (15.64 ± 6.55%)	Propionibacteriaceae sp. OTU88 (10.13 ± 12.85%)	*Undibacterium* OTU173 (14.65 ± 29.15%)	Chloroplast sp. OTU481 (3.73 ± 6.81%)	*Ralstonia pickettii* OTU502 (9.82 ± 9.66%)
Sphingomonas aquatilis OTU488 (5.50 ± 1.20%)	*Methyloparacoccus* OTU14 (5.77 ± 7.35%)	*Undibacterium* OTU230 (13.67 ± 26.17%)	Comamonadaceae sp. OTU235 (3.00 ± 3.39%)	Comamonadaceae sp. OTU486 (9.74 ± 8.52%)
Ralstonia pickettii OTU502 (4.44 ± 4.07%)	*Paeniclostridium* OTU107 (4.87 ± 5.25%)	*Undibacterium* OTU135 (4.93 ± 8.31%)	*Novosphingobium* OTU331 (2.44 ± 0.84%)	*Pelomonas* OTU496 (7.74 ± 8.36%)
Methylobacterium-Methylorubrum OTU491 (3.32 ± 1.26%)	*Candidatus Competibacter* OTU81 (4.61 ± 3.82%)	*Paeniclostridium* OTU107 (4.63 ± 6.05%)	*Cetobacterium* OTU126 (2.23 ± 0.83%)	*Sphingomonas aquatilis* OTU488 (5.44 ± 4.63%)
Pelomonas OTU496 (3.20 ± 3.59%)	HOC36 sp. OTU15 (4.50 ± 4.45%)	*Anoxybacillus* OTU202 (3.39 ± 5.96%)	Comamonadaceae sp. OTU471 (2.01 ± 0.78%)	*Methylobacterium-Methylorubrum* OTU491 (2.68 ± 1.05%)
Amnibacterium OTU161 (2.20 ± 0.63%)	HOC36 sp. OTU65 (3.72 ± 3.93%)	*Thermus* OTU194 (3.23 ± 5.05%)	HOC36 sp. OTU478 (1.99 ± 1.55%)	*Roseomonas gilardii* OTU497 (1.87 ± 0.90%)
2013Ark19i OTU156 (1.29 ± 1.04%)	*Escherichia-Shigella* OTU206 (3.57 ± 4.13%)	*Cetobacterium* OTU223 (3.21 ± 6.32%)	Comamonadaceae sp. OTU367 (1.90 ± 2.74%)	*Amnibacterium* OTU161 (1.54 ± 1.12%)
Roseomonas gilardii OTU497 (0.99 ± 0.47%)	*Cetobacterium* OTU126 (3.46 ± 4.91%)	*Romboutsia* OTU228 (3.11 ± 5.98%)	Chloroplast sp. OTU458 (1.89 ± 2.83%)	*Staphylococcus* OTU499 (1.19 ± 0.25%)

Table S5. The Nearest Sequenced Taxon Index (NSTI) values of KEGG function prediction based on PICRUSt2 at five different sites. The accuracy of the functional predictions is decreased with increasing NSTI value. The same letter indicates no significant differences ($p > 0.05$) between the two groups, while the different letter indicates significant differences ($p < 0.05$ or $p < 0.01$) between the two groups.

Group	Mean NSTI value
G	0.0083 ± 0.0020 a
C	0.1869 ± 0.0615 b
M	0.0525 ± 0.0361 a
S	0.2432 ± 0.0193 b
W	0.0064 ± 0.0003 a
